# Subcellular Localization of Monoglucosyldiacylglycerol Synthase in *Synechocystis* sp. PCC6803 and Its Unique Regulation by Lipid Environment

**DOI:** 10.1371/journal.pone.0088153

**Published:** 2014-02-06

**Authors:** Tiago Toscano Selão, Lifang Zhang, Candan Ariöz, Åke Wieslander, Birgitta Norling

**Affiliations:** 1 School of Biological Sciences, Nanyang Technological University, Singapore, Singapore; 2 Department of Biochemistry and Biophysics, Arrhenius Laboratories, Stockholm University, Stockholm, Sweden; University of Hyderabad, India

## Abstract

Synthesis of monogalactosyldiacylglycerol (GalDAG) and digalactosyldiacylglycerol (GalGalDAG), the major membrane lipids in cyanobacteria, begins with production of the intermediate precursor monoglucosyldiacylglycerol (GlcDAG), by monoglucosyldiacylglycerol synthase (MGS). In *Synechocystis* sp. PCC6803 (*Synechocystis*) this activity is catalyzed by an integral membrane protein, Sll1377 or MgdA. *In silico* sequence analysis revealed that cyanobacterial homologues of MgdA are highly conserved and comprise a distinct group of lipid glycosyltransferases. Global regulation of lipid synthesis in *Synechocystis* and, more specifically, the influence of the lipid environment on MgdA activity have not yet been fully elucidated. Therefore, we purified membrane subfractions from this organism and assayed MGS activity *in vitro*, with and without different lipids and other potential effectors. Sulfoquinovosyldiacylglycerol (SQDAG) potently stimulates MgdA activity, in contrast to other enzymes of a similar nature, which are activated by phosphatidylglycerol instead. Moreover, the final products of galactolipid synthesis, GalDAG and GalGalDAG, inhibited this activity. Western blotting revealed the presence of MgdA both in plasma and thylakoid membranes, with a high specific level of the MgdA protein in the plasma membrane but highest MGS activity in the thylakoid membrane. This discrepancy in the subcellular localization of enzyme activity and protein may indicate the presence of either an unknown regulator and/or an as yet unidentified MGS-type enzyme. Furthermore, the stimulation of MgdA activity by SQDAG observed here provides a new insight into regulation of the biogenesis of both sulfolipids and galactolipids in cyanobacteria.

## Introduction

Proposed by the endosymbiotic theory to be the ancestors of plant chloroplasts, cyanobacteria are commonly used to investigate the biogenesis of the thylakoid membrane (TM). Two major ideas currently prevail, one considering cyanobacterial TM to be a physically separate entity and the other stating that this membrane has either permanent or transient contact with the plasma membrane (PM) [1]. Although much work on the synthesis of both proteins and pigments of the photosystems and their regulation has been performed with the unicellular cyanobacterium *Synechocystis* sp. PCC6803 (hereafter referred to as *Synechocystis*) [2]–[4], much less is known about the lipid components. If the TM and PM have no connection, both membranes might synthesize their own lipids. Alternatively, vesicular lipid transport, such as in chloroplasts [5], could occur between these membranes, although there is at present no direct evidence for this theory. On the other hand, if the membranes are connected, lipids synthesized by either or both could flow between them. Accordingly, a better understanding of lipid synthesis and its regulation in cyanobacteria is central to understanding the process of thylakoid biogenesis.

Although the major lipid constituents of membranes in most non-photosynthetic organisms are phospholipids, the major membrane lipids of chloroplasts and cyanobacteria do not contain a phosphorus (P) atom, possibly because both plants and many bacterial species have limited access to in-organic phosphorus (P_i_) [6]–[9]. Monogalactosyldiacylglycerol (GalDAG: 1,2-diacyl-3-*O*-(β-D-galactopyranosyl)-*sn*-glycerol), one of the most abundant membrane lipids in the biosphere, often constitutes 50 mol% or even more of the total lipid content of cellular (or chloroplast) membranes in photosynthetic organisms [10], [11]. Not only does GalDAG play an important structural role in membranes but it is also vital for the correct functioning of the photosynthetic complexes and pathways [12].

The pathways by which GalDAG is synthesized in plant chloroplasts and cyanobacteria are quite distinct. In chloroplasts, MGD1 catalyses the reaction of *sn*-1,2-diacylglycerol with UDP-activated galactose (UDP-1-α-galactose) [13] whereas in cyanobacteria diacylglycerol is first combined with UDP-glucose by monoglucosyldiacylglycerol synthase (or MGS) to yield the precursor monoglucosyldiacylglycerol (GlcDAG: 1,2-diacyl-3-*O*-(β-D-glucopyranosyl)-*sn*-glycerol) [14]. In *Synechocystis* MGS is encoded by the gene *sll1377* or *mgdA* [15], [16] and deletion of this gene renders cells non-viable [15]. GlcDAG does not accumulate in *Synechocystis* cells under normal growth conditions, since its glucose moiety is rapidly converted into galactose by an as yet uncharacterized epimerase, producing GalDAG [14]. GalDAG is the acceptor substrate for formation of the other major galactolipid, digalactosyldiacylglycerol (GalGalDAG: 1,2-diacyl-3-*O*-[α-D-galactopyranosyl-(1→6)-*O*-β-D-galactopyranosyl]-*sn*-glycerol) through a pathway thought to be conserved from cyanobacteria to plants [17].

The various homologues of MGS can potentially be regulated both transcriptionally and posttranslationally, but the lipid environment is a major regulatory factor of this enzymatic activity. In organisms such as *Acholeplasma laidlawii, Streptococcus pneumoniae*, *Mycoplasma pneumoniae* and *M. genitalium* GlcDAG synthesis is dependent on the presence of an activator lipid, the anionic phosphatidylglycerol (PG) [18]–[21] Enzymatic activity is stimulated further by the presence of small amounts (up to 10 mol%) of phosphatidylethanolamine (PE), possibly due to increase of the spontaneous curvature of the bilayer when this zwitterionic lipid, which itself is prone not to form a bilayer, is present [18].

The MGS enzymes of *A. laidlawii* and *S. pneumoniae* are monotopic membrane proteins, whereas their counterparts in *Synechocystis* and *M. pneumoniae* [22] are predicted to be integral membrane proteins containing several transmembrane helices. Therefore, regulation of MgdA may differ from that for other enzymes with similar activities. To further understand how sugar lipid synthesis is regulated in *Synechocystis*, we have examined the localization of MgdA and its response to different lipid species in this organism. Both *in vitro* and *in vivo* measurements indicated that the regulation of *Synechocystis* MgdA by its lipid environment differs from that of other types of MGS enzymes. The steps involved in the synthesis of GlcDAG and GalDAG were found to occur in all internal membranes. Moreover, *in silico* sequence analysis revealed that homologues of MgdA in cyanobacteria are highly conserved. Based on our findings, a model for the biogenesis of cyanobacterial membranes is presented.

## Materials and Methods

### Media and Cell Growth

*Synechocystis* sp. 6803 was grown in glass bottles with 1 L BG11 medium [23] at 30°C, bubbling with sterile air and at a light intensity of 50 µE sec^−1^ m^−2^. Cell cultures with an OD_730_ 1–1.5 were harvested by centrifugation (8,000 g, 4°C, 15 minutes), washed once in cold 20 mM potassium phosphate buffer (pH 7.5) and cell pellets were stored at −80°C. *E. coli* C41 (DE3) was routinely grown in LB medium (liquid or solid) for cloning and in 2× LB medium for enzyme overexpression, both supplemented with 50 µg mL^−1^ kanamycin.

### Fractionation of *Synechocystis* Membranes

The pellets obtained as described above were washed once again with chilled lysis buffer (20 mM potassium phosphate buffer, pH 7.5), pelleted and resuspended in 1 mL lysis buffer containing protease inhibitors (Complete, EDTA-free, Roche). This suspension was then lysed by vortexing vigorously in the presence of 0.17–0.18 mm glass beads (Sartorius). Sucrose gradients were performed and aqueous two-phase partitioning carried out according to previously established protocols [24], with these modifications. Two-phase systems were incubated on ice for 15 minutes between centrifugations and thoroughly mixed by inversion in closed ice box. This procedure yields “light” (low-density) plasma membrane (PM1, less than 200 µg protein/L culture with an OD_730_ = 1) and “heavy” (high-density) plasma membrane (PM2, roughly 2–3 mg protein/L culture), as well as purified TM (approximately 20 mg protein/L culture). PM1 separates in an orange band between 10 and 30% (w/w) borders in the sucrose gradient, while PM2 and TM are isolated from the 38–42% (w/w) region of this gradient using aqueous two-phase partitioning [24]. Protein was quantified by the Peterson procedure [25] and samples stored at −80°C for further use.

### Protein Electrophoresis and Western Blotting

To assess purity, 5 µg protein from each fraction was solubilized in SDS-PAGE sample buffer supplemented with 7M urea, incubated for 30 minutes at room temperature and eletrophoresed in 12% TGX precast SDS-PAGE gels (BioRad). The gels were then transferred to PVDF membranes, which were subsequently probed with antibodies for characteristic TM (PsaA and CP43, Agrisera) and PM proteins (KtrE, obtained from Nobuyuki Uozumi and PixJ1, peptide antibody [4]), as well as for MgdA (a kind gift from Mie Shimojima). The blots were developed using an anti-rabbit antibody conjugated with horseradish peroxidase (GE Healthcare), enhanced chemiluminescence (ECL Prime, GE Healthcare) and a CCD camera (LAS-4000 Fujifilm), in the “Precision” mode and with automatic exposure time. For comparison of the migration of the bands detected to that of the prestained markers (PageRuler Plus, Thermo Scientific), blot images were merged with the MultiGauge software (v. 3.2, Fujifilm). Each individual blot was performed in triplicate.

### Cloning MgdA Variants

Truncated MgdA constructs were generated in order to examine the function of each individual transmembrane region. All primers consisted of a gene-specific annealing sequence of approximately 20 bp and common overhangs containing the recombination sites (see Table S1). The major vector employed was pNIC28-Bsa4 (GenBank accession EF198106), developed by Posher Gileadi at SGC Oxford. All targets were fused with an N- or C-terminal hexahistidine tag to simplify purification, as well as a cleavage site for TEV protease to facilitate removal of this tag. Ligation independent cloning (LIC) was applied to all variants, in accordance with published procedures [26], [27]. In brief, a RecA^–^ strain of *E. coli* was utilized for the initial cloning and plasmid production, followed by re-transformation of the plasmid containing the target gene into the expression strain (a T1-phage resistant BL21-DE3 Rosetta strain developed in-house at the Protein Production and Purification Platform, SBS/NTU).

### Expression of MgdA Variants in *E. coli*

For expression of MgdA variants, single colonies from LB-agar plates were grown overnight in 5 mL 2× LB medium supplemented with 50 µg.mL^−1^ kanamycin at 37°C, shaking at 200 RPM. The following morning, 2 mL of the starter culture was inoculated into 100 mL fresh 2× LB medium containing antibiotic and grown for another 2 hours, after which the temperature was reduced to 22°C and the cultures incubated for an additional 60 minutes. Expression was induced by addition of 1 mM isopropyl β-D-1-thiogalactopyranoside (IPTG) and the cells allowed to grow for 22 hours at 22°C with shaking. Cultures were harvested by centrifugation (3,000 g, 4°C, 15 minutes), washed in wash buffer (100 mM HEPES with 20 mM MgCl_2_, pH 8.0), resuspended to an OD_600_ = 40 in the assay buffer (100 mM HEPES, 20 mM MgCl_2_ and 20 mM CHAPS, pH 8.0) and sonicated three times for 5 minutes each in a water-bath sonicator (Fisherbrand) to obtain whole cell lysates, which were stored in aliquots at −80°C until further use.

For large-scale expression of full-length MgdA, 500-mL cultures were induced as described above and total membrane isolated as follows: after washing in wash buffer supplemented with Complete EDTA-Free protease inhibitors (Roche Applied Science), the cells were homogenized (four passes at 12,000 psi), debris removed by slow-speed centrifugation (3,000 g, 4°C, 15 minutes) and the total membrane fraction then obtained by high-speed centrifugation (200,000 g, 4°C, 45 minutes). These fractions were resuspended in storage buffer (wash buffer supplemented with 10% glycerol) and kept in aliquots at −80°C. Protein content were quantified by the Peterson method [25].

### Assay of MGS Activity and Thin-layer Chromatography (TLC)

MGS activity was assayed as described previously [18], with certain modifications. In brief, either 600 µg total membrane protein or 200 µg protein of the different *Synechocystis* membrane fractions was washed once with wash buffer, centrifuged (250,000 g, 4°C, 20 minutes) and resuspended in 100 µL of assay buffer. These suspensions were subsequently incubated on ice for 30 min, sonicated in a water-bath sonicator (1 minute, 3 times) and combined with 80 µL of mixed lipid micelles (Avanti Polar Lipids) [18], followed by additional 30 minutes of incubation on ice. 10 µL of UDP-[^14^C]glucose solution (Perkin-Elmer, 0.02 µCi.µL^−1^, 302 µCi.µmol^−1^, diluted 1∶5) was then added and incubation continued for another 30 minutes at 30°C, after which the reaction was stopped by adding 390 µL chloroform:methanol (1∶2) and lipid phases extracted according to Bligh and Dyer [28], [29]. Thereafter, these lipid phases were vacuum-dried, redissolved in 40 µL chlorophorm:methanol (2∶1) and loaded onto an aluminum-backed TLC plate (Merck), which was developed with a chloroform:methanol:28% NH_4_OH system (78∶42∶6). After TLC run, the plate was dried and exposed at least 22 hours on a Kodak Imaging Screen K (Kodak) and analyzed with a Typhoon scanner (Typhoon Trio, GE Healthcare) using standard settings for autoradiography. Activity was calculated based on the intensity of the radioactive GlcDAG band in comparison to serial dilutions standard of UDP-[^14^C] glucose, employing the ImageQuant TL software (v. 7.0, GE Healthcare).

Variants expressed in *E. coli* were assayed with this same protocol using 100 µL of culture at OD_600_ = 40. Full-length MgdA recombinant was assayed using 200 µg total *E. coli* membrane suspension in 100 µL MGS assay buffer.

### Pulse-labeling of Newly Synthesized Lipids in *Synechocystis*

*Synechocystis* cultures (at OD_730_ = 1.5) were centrifuged, cell pellets were washed three times with fresh BG11 and then divided into 4 individual cultures in 100 mL E-flasks (final OD_730_ of 15 in 30 mL of BG11). Following 2 hours of incubation at 30°C with air bubbling, acyl chains were labeled by addition of 12 µCi [^14^C]-acetate (Perkin-Elmer) to each culture (corresponding to a final concentration of 7.6 µM), which can be taken up rapidly by the cells. Then, these mixtures were incubated at 30°C with air bubbling and at the time intervals indicated, labeling was stopped by addition of 1 mL of 1 M sodium acetate (pH = 7.5) to each culture. Cells were transferred to a 50-mL Falcon tube, cooled rapidly in liquid nitrogen to slow down metabolism and centrifuged (15 minutes at 8,000 g, 4°C). The resulting pellets were flash-frozen in liquid nitrogen and stored at −80°C, until preparation of total membrane, PM1, PM2 and TM fractions as described above. 5,000 CPM from the total membrane fraction at each time point and 2,000 CPM from each isolated fraction were subjected to TLC and analyzed as described above.

### *In silico* Analysis

Transmembrane helices were predicted using TMHMM 2.0 (www.cbs.dtu.dk/services/TMHMM), TOPCONS (http://octopus.cbr.su.se), ΔG Prediction server (http://dgpred.cbr.su.se/), BioInfoBank MetaServer (http://meta.bioinfo.pl) and HHPred (http://toolkit.tuebingen.mpg.de/hhpred), at the default setting.

## Results and Discussion

### Subcellular Localization of MgdA Protein and Activity in *Synechocystis*

In plant chloroplasts, lipid synthesis occurs exclusively in the two envelope membranes, rather than in the intraorganellar TM. Therefore, formation of the TM should be entirely dependent on lipid transport from these envelopes, most likely in the form of lipid vesicles [30]. To localize synthesis of the galactolipid precursor GlcDAG in *Synechocystis*, we investigated the distribution of both MgdA protein and activity in the different membranes.

**Figure 1:**
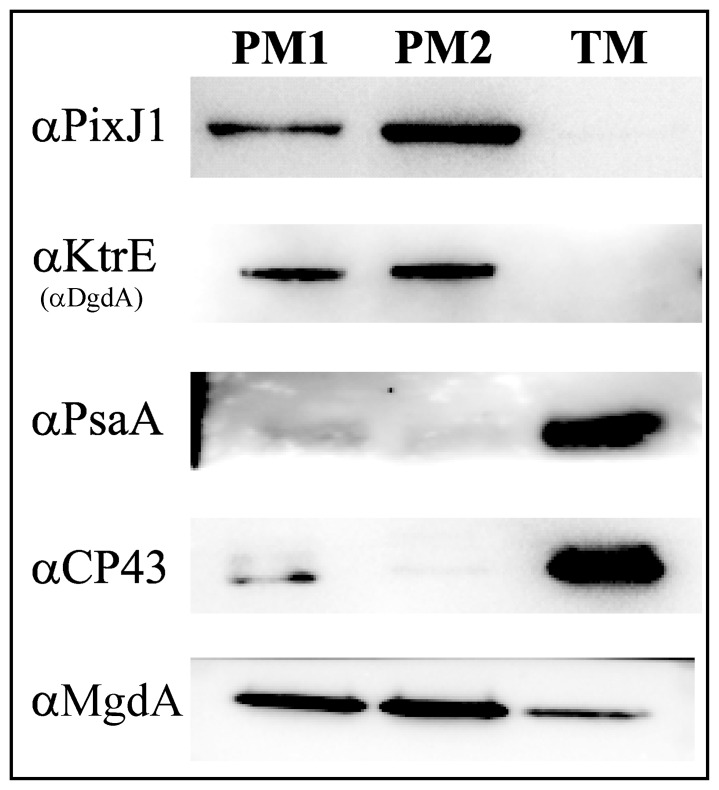
Western blotting analysis of membrane subfractions isolated from *Synechocystis*. Each gel lane was loaded with 5 µg membrane protein and probed with the antibodies indicated. PM1– low-density plasma membrane; PM2– high-density plasma membrane; TM – thylakoid membrane. All individual blots were performed in triplicate, using independent samples. For further details, please see the Materials and Methods.

The purity of the *Synechocystis* membrane fractions isolated by sucrose gradient and two-phase partition [24] was confirmed by western blots (Figure 1). The marker proteins for the TM (CP43 and PsaA) only exist in TM fraction, while PM marker proteins (PixJ1 [4] and KtrE (also known as DgdA [31])) are only present in PM1 and PM2 fractions. In comparison to the unambiguous distribution of these markers, MgdA was detected in both PM1 and PM2, as well as in TM, although the amount present in TM was significantly lower (Fig. 1).

**Figure 2:**
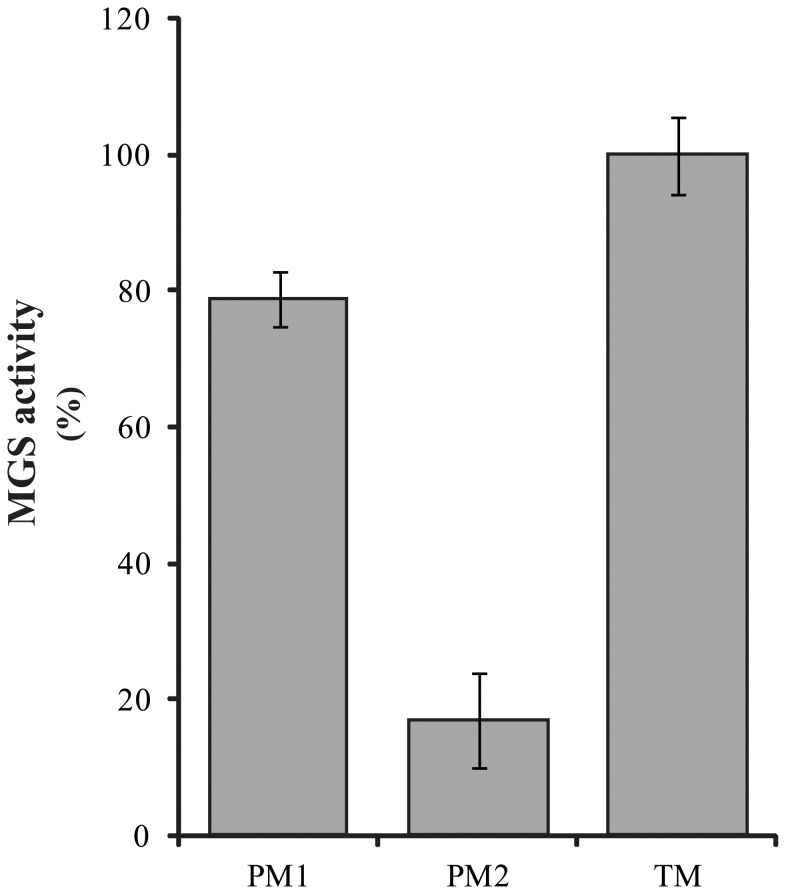
MGS activity in different membrane subfractions isolated from *Synechocystis*. The values shown are averages of three independent experiments, using 200 µg protein per assay. 100% activity corresponds to 23.8±1.4 pmol UDP-glucose incorporated.h^−1^.mg total protein^−1^. PM1– low-density plasma membrane; PM2– high-density plasma membrane; TM – thylakoid membrane. Error bars indicate standard deviations.

To our surprise, the TM fraction exhibited the highest MGS activity, followed by the PM1 and the PM2 fractions (Fig. 2). Previously, Omata and Murata [32] reported that “lighter”, “cytoplasmic” membranes isolated from *Synechococcus* on a sucrose gradient demonstrated higher MGS activity than “heavier” membranes, which is consistent with our present findings, since the PM1 fraction should correspond to their lighter fraction. Moreover, using our aqueous two-phase system we were able to further separate their “heavy”, “thylakoid” fraction into the TM and PM2 fractions (as shown in [24]) and found that the highest specific activity was present in TM (Fig. 2). Accordingly, the lower specific activity observed by Omata and Murata for the “thylakoid membrane” fractions [32] may be due to the simultaneous presence of PM2 (with low specific activity) in the same fraction as TM.

### Localization of *de novo* Lipid Synthesis in *Synechocystis* Membranes

**Figure 3:**
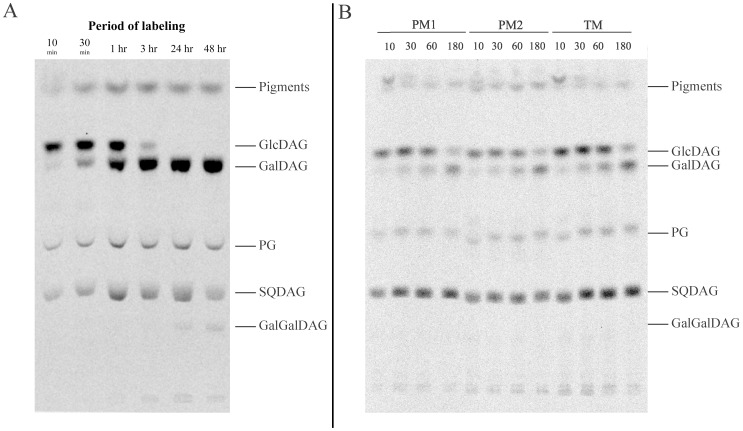
TLC analysis of radioactive acyl chains following pulse-chase labeling of lipids in *Synechocystis* membrane fractions with [^14^C]-acetate. **A.** Autoradiogram of labeling of total membrane lipids. All lanes contained 5000**B.** Autoradiogram of lipid labeling in the different membrane subfractions. All lanes contained 2000 CPM labeled lipids. The time after addition of radioactive acetate is given in minutes. PM1– low-density plasma membrane; PM2– high-density plasma membrane; TM – thylakoid membrane.

When examining *de novo* lipid synthesis in *Synechocystis* membranes by labeling acyl chains with [^14^C]-acetate, it was necessary to label for at least 10 minutes (Figure S1). Within 10 minutes, selectively labeled GlcDAG had already accumulated in the total membrane fraction; during the following 3 hours, over 90% of this labeled precursor was converted into GalDAG, while, surprisingly, labeling of GalGalDAG required more than 24 hours (Fig. 3A). The relatively rapid biosynthesis of GlcDAG and its conversion to GalDAG highlight the indispensability of these lipids in cyanobacteria. It was shown previously that deletion of the enzyme responsible for synthesis of GalGalDAG (DgdA) exerts only minor effects on cell growth and photosynthesis reactions [17] and its slow rate of synthesis here also supports a less critical role for this lipid in *Synechocystis*. The non-sugar lipids PG and SQDAG were also labeled, even at the earliest time-points, and the level of this labeling appeared to remain rather constant.

In experiments designed to further clarify the localization of GlcDAG synthesis, purified PM (PM1 and PM2) and TM fractions from the pulse-labeled cells were all found to contain radio-labeled GlcDAG even at the first time-point (10 minutes) tested after addition of [^14^C]-acetate (Fig. 3B). This observation is consistent with the presence of MGS activity in all these membranes (Fig. 2). Furthermore, the PM1, PM2 and TM fractions all epimerized GlcDAG to GalDAG. Therefore, it can be concluded that all of these membranes harbor the machinery required to synthesize GlcDAG and convert it to GalDAG.

### Influence of the Lipid Environment on MGS Activity

TM is the most abundant membrane fraction in most cyanobacterial cells and large amounts of sugar lipids are required as building blocks for its biogenesis. This explains the necessity for higher glucosyltransferase activity in the TM rather than PM2. However, the lower level of MgdA protein in the TM (Fig. 1) raises the question as to how sugar lipid synthesis is regulated in *Synechocystis*. Since the lipid composition of the membrane influences lipid glycosyltransferase (GT) activities in other organisms [18]–[21], [33], we tested this possibility here as well.

**Figure 4:**
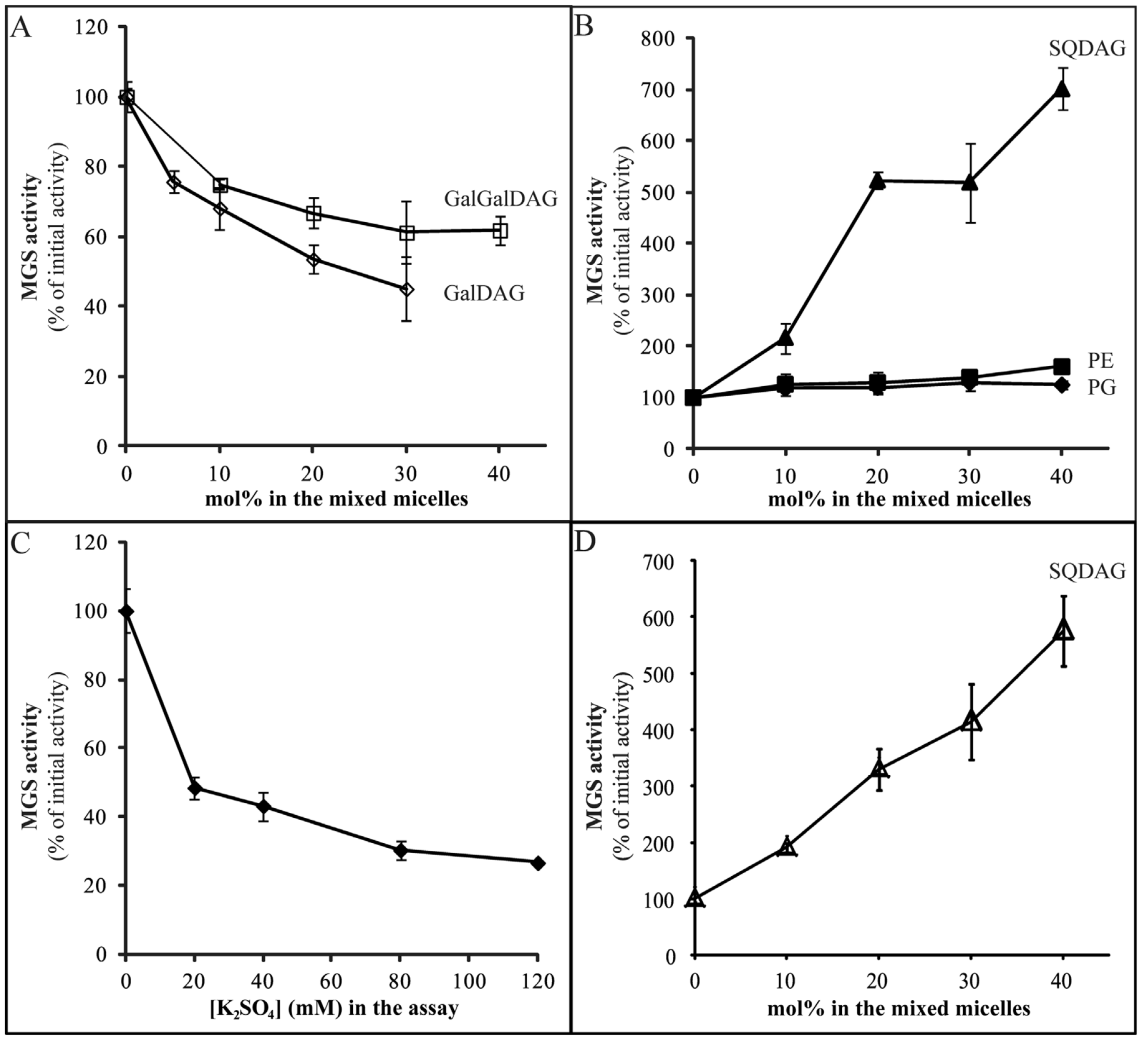
MGS activity in the presence of mixed micelles containing different amounts of various lipids and with different concentrations of K_2_SO_4_. The values shown are averages of four independent experiments, using 600 µg total membrane protein from two independent cultures per assay. 100% activity corresponds to 3.8±0.17 pmol UDP-glucose incorporated.h^−1^.mg total protein^−1^. **A.** The influence of GalDAG (◊) and GalGalDAG (□) on MgdA activity. **B.** The influence of SQDG (▴), PE (▪) and PG (♦) on MgdA activity. **C.** The influence of sulfate ions (♦) on MGS activity in the presence of 30 mol% PG. **D.** Influence of SQDG (Δ) on recombinant full-length MgdA activity. 100% activity corresponds to 4.60±0.20 pmol UDP-glucose incorporated.h^−1^.mg total protein^−1^.

Under normal conditions GalDAG and GalGalDAG can account for approximately 50 and 26 mol%, respectively, of the total lipid in *Synechocystis* membranes [17]. However, the presence of either of these lipids in our mixed-micelle assay system at any of the concentrations tested lowered MgdA activity (Fig. 4A), with GalDAG exerting a more pronounced effect. We propose that this represents feedback inhibition. In *Synechocystis*, the pathways for sulfolipid and galactolipid synthesis share the same initial substrate (DAG) and must therefore be delicately balanced. Koichiro Awai and co-workers [17] have shown that when GalGalDAG synthesis is prevented (by a *dgdA* knock-out mutation), the amount of SQDAG in the membranes increases by 60% (from 14.3 to 22.5 mol%), while the level of GalDAG increases by only 20% (from 49.7 to 60.4 mol%). Together with our data, these observations indicate that regulation of these two synthetic pathways is closely coordinated.

PG and other anionic phospholipids stimulate the activity of several different lipid GTs in various organisms, such as *A. thaliana* [18], [19], [33] and *Homo sapiens* (unpublished observation) substantially. However, here PG exerted only a marginal stimulatory effect (25±8%) on MgdA activity at the maximal concentration tested (40 mol%) and was actually an entirely unnecessary component of the mixed micelle (Fig. 4B), in complete contrast to similar enzymes in other organisms (e.g., *A. laidlawii*). In *E. coli*, PG appears to be the major regulator of lipid synthesis, also controlling a key step in synthesis of PE, the most abundant lipid in this organism, by a mechanism involving surface charge and density [34]. Phosphatidylethanolamine (PE), which stimulates certain lipid GTs but is not found in the inner membranes of *Synechocystis*, also exhibited a mild activity-enhancing effect on MgdA, here 60±7%, at 40 mol% concentration (Fig. 4B).

The most potent activator of MgdA activity was a constituent of the photosynthetic membrane, SQDAG, which doubled this activity at a concentration of 10 mol% (Fig. 4B) and stimulated 7-fold at the highest concentration tested (40 mol%). This is the first time such pronounced stimulation of a lipid GT by SQDAG has been reported. The more detailed discussion will be elucidated in the later paragraphs.

### Membrane Curvature has Little Effect on MgdA Activity

The ratio between lipids that are prone to form bilayers or not influences membrane curvature and lipid phase equilibria, which in turn affect functional features of membrane proteins [18], [35], [36]. For example, the activity of lipid GTs is sensitive to membrane curvature and surface charge [18], [19], [21], [33], [37]. In addition, in the presence of anionic lipids MGS and DGS in *A. laidlawii* (alMGS and alDGS) are stimulated by the zwitterionic (neutral) PE and other nonbilayer-prone lipids [18], [35], [38].

**Figure 5:**
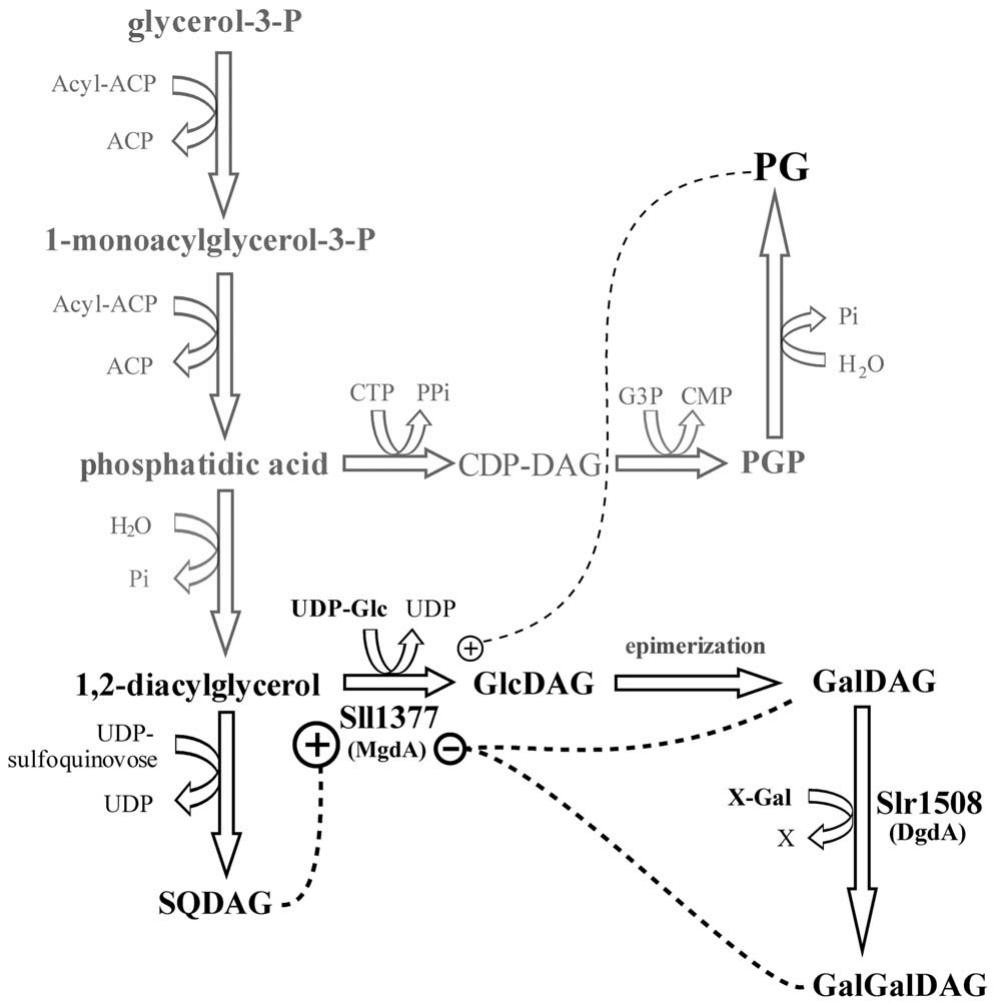
A simplified proposed scheme for lipid synthesis in *Synechocystis*. The semi-transparent text and arrows indicate synthesis of lipid precursors discussed in the text. The dashed lines represent putative regulatory interactions with MgdA. ACP – acyl carrier protein; G3P – glycerol-3-phosphate; CDP-DAG – CDP-diacylglycerol; Glc – glucose; Gal – galactose; PGP – phosphatidylglycerolphosphate.

Both GalDAG and GalGalDAG have neutral headgroups, but only the latter forms bilayers [36]. However, addition of either of these two major sugar lipids with different effects on membrane curvature impaired MgdA activity (Fig. 4A). Moreover, although GalDAG and PE both have neutral headgroups and are not prone to form bilayers, PE stimulated MgdA activity somewhat (Fig. 4A and B). Similarly, GalGalDAG and SQDAG, both of which form bilayers, exert opposite effects (Fig. 4A and B). It appears likely that in *Synechocystis* MgdA activity is not influenced by the bilayer curvature, in contrast to alMGS and alDGS in *A. laidlawii* [18], [35]. We suggest that, as final products of the pathway, GalDAG and GalGalDAG interact with and allosterically regulate MgdA (or a regulatory interacting partner). This would allow the cells to maintain the proper lipid composition of their membranes by reducing production of the precursor GlcDAG when sufficient galactolipids are already present (Fig. 5).

### SQDAG is a Potent and Specific Activator of *Synechocystis* MgdA

*Synechocystis* MgdA activity was specifically and very potently stimulated by SQDAG, a negatively charged lipid component of cyanobacterial membranes (Fig. 4B). In an attempt to understand the underlying mechanism, we performed an experiment similar to the one previously conducted with the *A. laidlawii* enzymes [40]. Since the SQDAG headgroup contains a sulfonic acid rather than phosphate moiety, MgdA activity was assayed in the presence of increasing concentrations of sulfate ions and unexpectedly, as shown in Fig. 4C, as little as 20 mM sulfate, in the presence of 30 mol% PG, decreased this activity by 52% and this suppression was even greater at higher concentrations of sulfate. Thus, the stimulation by SQDAG cannot be explained either by its negative surface or sulfate/sulfonic acid moiety.

When *mgdA* gene was expressed in *E. coli*, only a small amount of the recombinant enzyme was produced but the bacterial membranes exhibited potent MGS activity that was stimulated by SQDAG in the same manner as above (Fig. 4D). This observation provides strong support for the unique and probably specific interaction of SQDAG with MgdA. SQDAG contains a large polar head with a negative charge located some distance from the hydrophobic portion of the membrane. As sulfate alone did not activate MgdA, activation by SQDAG must be somehow dependent on the nature of the head group and/or of the entire molecule. It is noteworthy that lipids with a similar (sugar) head group, but no negative charge (such as GalDAG and GalGalDAG), were inhibitory (Fig. 4A).

Curiously, synthesis of SQDAG is not an absolute requirement for all cyanobacteria. *Synechocystis* strains lacking the *sqdB* gene require exogenous SQDAG for survival, whereas in *Synechococcus elongatus* sp. PCC7942 (*Synechococcus*) such a knockout produced no obvious effect on cell growth [39]. This difference might be explained by the recent observation that deletion of the *sqdB* gene affects DNA replication and cell division in *Synechocystis*, but not in *Synechococcus* [40].

We propose that this unique regulation of MgdA activity by SQDAG might be involved in maintaining lipid homeostasis in cyanobacteria, especially under stress conditions. For example, in the absence of inorganic phosphate, both *Synechocystis* and *Synechococcus* accumulate SQDAG and GalGalDAG at the expense of PG [41]. Such an exchange of phospholipids for sulfolipids has been suggested to play an important evolutionary role for picocyanobacteria, which must compete with heterotrophic bacteria for a limited supply of phosphate [42]. The situation is similar in freshwater environments [43]–[45] and *Synechocystis* can also take up and incorporate exogenous lipids such as SQDAG [43].

Control of lipid-synthesizing enzymes in both *Synechococcus* and *Synechocystis* must be at the post-translational, rather than the transcriptional level, since in neither organism is MgdA among the proteins whose expression is regulated by the SphS-SphR two-component system in response to inorganic phosphate [46], [47]. In fact, MgdA can be regulated post-translationally by temperature, both *in vivo* and *in vitro* [16], and, as our present results demonstrate (Fig. 4D), by SQDAG, GalDAG and GalGalDAG. As a freshwater cyanobacterium, *Synechocystis* is often subjected to restricted and/or fluctuating supplies of phosphate [7] and in such circumstances it may be more efficient to utilize a lipid species that does not contain phosphate, *i.e.*, SQDAG, to regulate lipid biosynthesis and/or as part of an effective rescue mechanism when little phosphate is available. A higher SQDAG concentration would then respond to inadequate levels of galactolipids by activating MgdA and vice-versa (Fig. 5). The level of SQDAG is also related to the availability of inorganic phosphate and, since the amount of galactolipid (namely GalGalDAG) is elevated during phosphate starvation [17], the synthesis of phospho-, sulfo- and galactolipids is probably coordinated in cyanobacteria by regulating the activity of appropriate enzymes.

However, the mechanism underlying this regulation on MgdA by SQDAG remains unknown. Several laboratories have observed direct interaction with or binding of SQDAG to different proteins *in vitro*, e.g., a protein that initiates replication in humans [48] and *E. coli* DNA polymerase I, which is inhibited by SQDAG [49]. In addition, this lipid stimulates protein import through the chloroplast outer envelope mediated by the precursor protein translocon [50]. To determine whether or not SQDAG regulates MgdA through direct interaction will require further experimental investigations.

### Conservation of the Transmembrane Helixes of MgdA among Cyanobacteria

**Figure 6:**
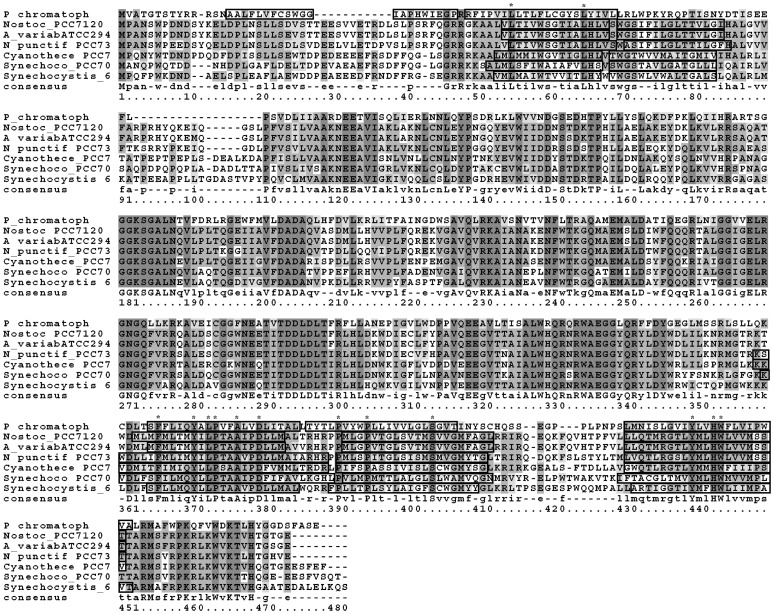
Conservation of transmembrane helices (TMH) among MGS homologues in cyanobacteria. ClustalW sequence alignments for several MGS homologues, with TMH (as predicted by the OCTOPUS server) highlighted by line boxes. These sequences were selected from a BlastP similarity search utilizing Sll1377 as the template and refer to enzymes from (top to bottom) *Paulinella chromatophora*, *Nostoc* sp. PCC7120, *Anabaena variabilis* ATCC29413, *Nostoc punctiforme* PCC73102, *Cyanothece* sp. PCC7424, *Synechococcus* sp. PCC7002 and *Synechocystis* sp. PCC6803.

Organisms related evolutionarily often retain specific features in the sequences and structures of their proteins and common regulatory mechanisms. Transmembrane domains are not only an important structural feature of proteins, but can also serve important regulatory roles. We chose typical model cyanobacteria, from single-cell to filamentous and both nitrogen-fixing and non-nitrogen-fixing species, to investigate conservation of sequence and secondary structure. When we analyzed the amino acid sequence of MgdA *in silico*, the 4 prediction programs employed all identified 5 putative transmembrane helices (TMH) that appear to be a conserved feature of cyanobacterial homologs (Fig. 6). In MgdA these are localized essentially to the N- and C-terminal regions (at residues TMH1 46–66, TMH2 68–88, TMH3 361–381, TMH4 389–409, and TMH5 435–455), flanking the putative catalytic domain and active site that face the cytoplasmic surface, with pronounced sequence similarity and remarkable conservation of these five transmembrane domains. Even in the case of the MGS homologue in the “chromatophore” inclusions of the amoeba *Paulinella chromatophora*, which are thought to be of cyanobacterial origin and the product of a recent endosymbiotic event [51], there is pronounced sequence similarity, with two TMH in the N-terminal and three in the C-terminal region (Fig. 6).

To evaluate the importance of different TMH segments in *Synechocystis* experimentally, several deletion variants were expressed in *E. coli* and assayed for activity. Even though many of these were expressed at a level higher than the full-length protein, none of the truncated constructs exhibited any glucosyltransferase activity, even in the presence of SQDAG or any other lipid activator (Fig. S5). In comparison, the full-length construct produced GlcDAG under the same conditions as those used for MgdA in *Synechocystis* (Fig. 4D). Thus, the transmembrane helices appear to be important for correct folding and/or enzymatic activity and may be one reason why MgdA responds preferentially to SQDAG, rather than PG, as do lipid GT enzymes without transmembrane domains.

### Regulation of the Synthesis of Sugar Lipids in *Synechocystis* – One Step Closer to the Answer?

**Figure 7:**
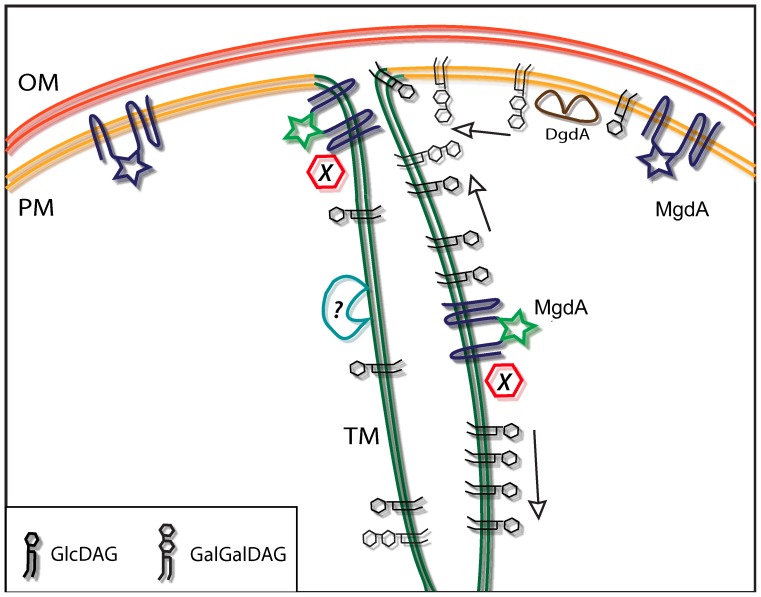
Working model for the biosynthesis and flow of sugar lipids between *Synechocystis* membranes. MgdA in the TM and in the putative connection between the TM and PM (PM1) is more active (symbolized by the green color of the star-shaped active site) than MgdA in PM2, due to the presence of an unknown regulator (indicated by “X”). In addition, there may be another, unknown MGS-type enzyme (indicated by a question mark) localized exclusively in the TM. DgdA is present exclusively in the PM. For simplicity, the unidentified sugar lipid epimerase has been omitted. The arrows represent lipid flows between membranes.

Our findings that the MgdA protein and activity are not distributed in the same manner among *Synechocystis* membranes could have two explanations (Fig. 7). First, an as yet unknown MGS-type enzyme located exclusively in the TM, may be responsible for the high activity observed there. As previously mentioned, deletion of the *sll1377* gene is lethal to *Synechocystis* [15] so such an enzyme, if it exists is unable to compensate for the loss of MgdA under these circumstances.

A second alternative could be the presence of a stimulator of MgdA activity in PM1 and TM. Such a regulator has already been suggested to enhance MgdA activity and thereby maintain membrane integrity under conditions of heat shock [52]. We attempted to isolate complexes containing MgdA, both by two-dimensional clear-native/SDS-PAGE electrophoresis, as well as by co-immunoprecipitation with the antibody against MgdA (Figs. S3 and S4). Regrettably, MgdA appeared to migrate alone in the gels and the antibody failed to pull-down putative MgdA-containing complexes, which may indicate that such a protein regulator does not exist or that its interaction with MgdA is not preserved during use of these approaches.

To examine whether reversible phosphorylation is involved in the regulation of MgdA, prior to addition of the enzymatic substrate we treated both PM2 and TM preparations with alkaline phosphatase in the presence of SQDAG. Since both treated and untreated preparations showed similar levels of activity (Fig. S2) this does not seem to be the case. Another possible explanation is that MgdA is regulated by a small molecule, as c-di-GMP regulates the GT enzymes PgaC and PgaD that synthesize the exopolysaccharides in *E. coli* [53].

The *Synechocystis* enzyme Slr1508 (DgdA) has GalGalDAG synthase activity *in vivo* [17] and deletion of the *dgdA* gene results in cells containing none of this lipid [17]. Strangely, this enzyme has also been reported to be part of a sodium-dependent potassium membrane transporter complex (Ktr) in *Synechocystis* [31], [54] and details concerning its reaction mechanism and the galactose donor are still unclear, since no assay for its *in vitro* activity is presently available. We detected the DgdA protein exclusively in PM (Fig. 1), implying that GalGalDAG synthesis should occur only at this location. This raises the question as to whether GalGalDAG is transported by vesicular traffic or by intermembrane diffusion through connections between the PM and TM, previously postulated to be PM1 [4]. While no consensus has been reached, accumulating evidence for a connection between the PM and TM [1], [3], [4], [55] supports lateral membrane diffusion (as hypothesized in Fig. 7).

### Concluding Remarks

Here, our initial observation of the discrepancies between the subcellular localization of protein and enzymatic activity involved in the crucial first step in galactolipid synthesis by *Synechocystis* motivated us to investigate the regulation of MgdA in greater detail. We have demonstrated that this MgdA has unique regulatory features, in particular the potent activation by sulfolipid SQDAG, which distinguishes it from other GlcDAG or GalDAG synthases. Pulse-labelling experiments with [^14^C]-acetate, as well as measurements of enzymatic activity revealed that GlcDAG and GalDAG are synthesized in both PM and TM, with highest specific activity in the latter. Whether MgdA is regulated post-translationally, by interaction with a small effector molecule or by a protein partner remains to be discovered. The possible existence of second MGS in *Synechocystis* and the interaction of SQDAG with MgdA are still open questions that will require further investigation.

## Supporting Information

Figure S1 TLC analysis of radioactive acyl chains following pulse-chase labeling of lipids in *Synechocystis* membrane fractions with [^14^C]-acetate.
(TIF)

Figure S2 Autoradiogram of the TLC analysis of MGS activity in the different *Synechocystis* membrane subfractions, in the presence or absence of calf intestinal alkaline phosphatase (CIAP).
(TIF)

Figure S3 Pulldown assays using MgdA antibodies and Protein A-agarose beads.
(TIF)

Figure S4 2D-clear native (CN)/SDS-PAGE gels and western blots of PM2 and TM.
(TIF)

Figure S5 TLC analysis of the MGS activity of recombinant MgdA variants.
(TIF)

Table S1 List of MGS constructs, the transmembrane helices (TMH) encoded by each construct and the primer sequences used for amplification.
(DOCX)

## References

[1] Stengel A, Gugel IL, Hilger D, Rengstl B, Jung H, et al (2012) Initial steps of photosystem II de novo assembly and preloading with manganese take place in biogenesis centers in *Synechocystis* . Plant Cell 24: 660–675. 22319052 10.1105/tpc.111.093914 PMC3315239

[2] Nixon PJ, Michoux F, Yu J, Boehm M, Komenda J (2010) Recent advances in understanding the assembly and repair of photosystem II. Ann Bot 106: 1–16. 20338950 10.1093/aob/mcq059 PMC2889791

[3] Rengstl B, Knoppova J, Komenda J, Nickelsen J (2012) Characterization of a *Synechocystis* double mutant lacking the photosystem II assembly factors YCF48 and Sll0933. Planta. 10.1007/s00425-012-1720-0 22847023

[4] Pisareva T, Kwon J, Oh J, Kim S, Ge C, et al (2011) Model for membrane organization and protein sorting in the cyanobacterium *Synechocystis* sp. PCC 6803 inferred from proteomics and multivariate sequence analyses. J Proteome Res 10: 3617–3631. 21648951 10.1021/pr200268r

[5] Westphal S, Soll J, Vothknecht UC (2001) A vesicle transport system inside chloroplasts. FEBS Lett 506: 257–261. 11602257 10.1016/s0014-5793(01)02931-3

[6] Dormann P, Benning C (2002) Galactolipids rule in seed plants. Trends Plant Sci 7: 112–118. 11906834 10.1016/s1360-1385(01)02216-6

[7] Schindler DW (1977) Evolution of phosphorus limitation in lakes. Science 195: 260–262. 17787798 10.1126/science.195.4275.260

[8] Geske T, Dorp KV, Dormann P, Holzl G (2012) Accumulation of glycolipids and other non-phosphorous lipids in *Agrobacterium tumefaciens* grown under phosphate deprivation. Glycobiology. 10.1093/glycob/cws124 22923441

[9] Nilsson L, Muller R, Nielsen TH (2010) Dissecting the plant transcriptome and the regulatory responses to phosphate deprivation. Physiol Plant 139: 129–143. 20113436 10.1111/j.1399-3054.2010.01356.x

[10] Aronsson H, Schottler MA, Kelly AA, Sundqvist C, Dormann P, et al (2008) Monogalactosyldiacylglycerol deficiency in *Arabidopsis* affects pigment composition in the prolamellar body and impairs thylakoid membrane energization and photoprotection in leaves. Plant Physiol 148: 580–592. 18641085 10.1104/pp.108.123372 PMC2528128

[11] Hartel H, Dormann P, Benning C (2000) DGD1-independent biosynthesis of extraplastidic galactolipids after phosphate deprivation in *Arabidopsis* . Proc Natl Acad Sci USA 97: 10649–10654. 10973486 10.1073/pnas.180320497 PMC27079

[12] Mizusawa N, Wada H (2012) The role of lipids in photosystem II. Biochim Biophys Acta 1817: 194–208. 21569758 10.1016/j.bbabio.2011.04.008

[13] Benning C, Ohta H (2005) Three enzyme systems for galactoglycerolipid biosynthesis are coordinately regulated in plants. J Biol Chem 280: 2397–2400. 15590685 10.1074/jbc.R400032200

[14] Sato N, Murata N (1982) Lipid biosynthesis in the blue-green alga (cyanobacterium), *Anabaena variabilis*. III. UDP-glucose:diacylglycerol glucosyltransferase activity in vitro. Plant Cell Physiol 23: 1115–1120.

[15] Awai K, Kakimoto T, Awai C, Kaneko T, Nakamura Y, et al (2006) Comparative genomic analysis revealed a gene for monoglucosyldiacylglycerol synthase, an enzyme for photosynthetic membrane lipid synthesis in cyanobacteria. Plant Physiol 141: 1120–1127. 16714404 10.1104/pp.106.082859 PMC1489894

[16] Shimojima M, Tsuchiya M, Ohta H (2009) Temperature-dependent hyper-activation of monoglucosyldiacylglycerol synthase is post-translationally regulated in *Synechocystis* sp. PCC 6803. FEBS Lett 583: 2372–2376. 19549521 10.1016/j.febslet.2009.06.033

[17] Awai K, Watanabe H, Benning C, Nishida I (2007) Digalactosyldiacylglycerol is required for better photosynthetic growth of *Synechocystis* sp. PCC6803 under phosphate limitation. Plant Cell Physiol 48: 1517–1523. 17932115 10.1093/pcp/pcm134

[18] Dahlqvist A, Nordstrom S, Karlsson OP, Mannock DA, McElhaney RN, et al (1995) Efficient modulation of glucolipid enzyme activities in membranes of *Acholeplasma laidlawii* by the type of lipids in the bilayer matrix. Biochemistry 34: 13381–13389. 7577924 10.1021/bi00041a015

[19] Karlsson OP, Dahlqvist A, Wieslander A (1994) Activation of the membrane glucolipid synthesis in *Acholeplasma laidlawii* by phosphatidylglycerol and other anionic lipids. J Biol Chem 269: 23484–23490. 8089114

[20] Andres E, Martinez N, Planas A (2011) Expression and characterization of a *Mycoplasma genitalium* glycosyltransferase in membrane glycolipid biosynthesis: potential target against mycoplasma infections. J Biol Chem 286: 35367–35379. 21835921 10.1074/jbc.M110.214148 PMC3195587

[21] Edman M, Berg S, Storm P, Wikstrom M, Vikstrom S, et al (2003) Structural features of glycosyltransferases synthesizing major bilayer and nonbilayer-prone membrane lipids in *Acholeplasma laidlawii* and *Streptococcus pneumoniae* . J Biol Chem 278: 8420–8428. 12464611 10.1074/jbc.M211492200

[22] Klement ML, Ojemyr L, Tagscherer KE, Widmalm G, Wieslander A (2007) A processive lipid glycosyltransferase in the small human pathogen *Mycoplasma pneumoniae*: involvement in host immune response. Mol Microbiol 65: 1444–1457. 17697098 10.1111/j.1365-2958.2007.05865.x

[23] Allen MM (1968) Simple conditions for growth of unicellular blue-green algae on plates. J Phycol 4: 1–4. 10.1111/j.1529-8817.1968.tb04667.x 27067764

[24] Huang F, Parmryd I, Nilsson F, Persson AL, Pakrasi HB, et al (2002) Proteomics of *Synechocystis* sp. strain PCC 6803: identification of plasma membrane proteins. Mol Cell Proteomics 1: 956–966. 12543932 10.1074/mcp.m200043-mcp200

[25] Peterson GL (1977) A simplification of the protein assay method of Lowry et al. which is more generally applicable. Anal Biochem 83: 346–356. 603028 10.1016/0003-2697(77)90043-4

[26] Aslanidis C, Dejong PJ (1990) Ligation-independent cloning of PCR products (LIC-PCR). Nucleic Acids Res 18: 6069–6074. 2235490 10.1093/nar/18.20.6069 PMC332407

[27] Graslund S, Sagemark J, Berglund H, Dahlgren LG, Flores A, et al (2008) The use of systematic N- and C-terminal deletions to promote production and structural studies of recombinant proteins. Protein Expr Purif 58: 210–221. 18171622 10.1016/j.pep.2007.11.008

[28] Bligh EG, Dyer WJ (1959) A rapid method of total lipid extraction and purification. Can J Biochem Physiol 37: 911–917. 13671378 10.1139/o59-099

[29] Sheng J, Vannela R, Rittrnann BE (2011) Evaluation of methods to extract and quantify lipids from *Synechocystis* PCC 6803. Bioresour Technol 102: 1697–1703. 20739178 10.1016/j.biortech.2010.08.007

[30] Andersson MX, Kjellberg JM, Sandelius AS (2001) Chloroplast biogenesis. Regulation of lipid transport to the thylakoid in chloroplasts isolated from expanding and fully expanded leaves of pea. Plant Physiol 127: 184–193. 11553746 10.1104/pp.127.1.184 PMC117974

[31] Zulkifli L, Akai M, Yoshikawa A, Shimojima M, Ohta H, et al (2010) The KtrA and KtrE subunits are required for Na^+^-dependent K^+^ uptake by KtrB across the plasma membrane in *Synechocystis* sp. strain PCC 6803. J Bacteriol 192: 5063–5070. 20656904 10.1128/JB.00569-10 PMC2944510

[32] Omata T, Murata N (1986) Glucolipid synthesis activities in cytoplasmic and thylakoid membranes from the cyanobacterium *Anacystis nidulans* . Plant and Cell Physiology 27: 485–490.

[33] Dubots E, Audry M, Yamaryo Y, Bastien O, Ohta H, et al (2009) Activation of the chloroplast monogalactosyldiacylglycerol synthase MGD1 by phosphatidic acid and phosphatidylglycerol. J Biol Chem 285: 6003–6011. 20023301 10.1074/jbc.M109.071928 PMC2825394

[34] Shibuya I (1992) Metabolic regulations and biological functions of phospholipids in *Escherichia coli* . Prog Lipid Res 31: 245–299. 1287667 10.1016/0163-7827(92)90010-g

[35] Vikstrom S, Li L, Karlsson OP, Wieslander A (1999) Key role of the diglucosyldiacylglycerol synthase for the nonbilayer-bilayer lipid balance of *Acholeplasma laidlawii* membranes. Biochemistry 38: 5511–5520. 10220338 10.1021/bi982532m

[36] Lindblom G, Brentel I, Sjolund M, Wikander G, Wieslander A (1986) Phase equilibria of membrane lipids from *Acholeplasma laidlawii*: importance of a single lipid forming nonlamellar phases. Biochemistry 25: 7502–7510. 3801429 10.1021/bi00371a037

[37] Dahlqvist A, Andersson S, Wieslander A (1992) The enzymatic synthesis of membrane glucolipids in *Acholeplasma laidlawii* . Biochim Biophys Acta 1105: 131–140. 1533160 10.1016/0005-2736(92)90171-h

[38] Vikstrom S, Li L, Wieslander A (2000) The nonbilayer/bilayer lipid balance in membranes. Regulatory enzyme in *Acholeplasma laidlawii* is stimulated by metabolic phosphates, activator phospholipids, and double-stranded DNA. J Biol Chem 275: 9296–9302. 10734070 10.1074/jbc.275.13.9296

[39] Aoki M, Sato N, Meguro A, Tsuzuki M (2004) Differing involvement of sulfoquinovosyl diacylglycerol in photosystem II in two species of unicellular cyanobacteria. Eur J Biochem 271: 685–693. 14764084 10.1111/j.1432-1033.2003.03970.x

[40] Aoki M, Tsuzuki M, Sato N (2012) Involvement of sulfoquinovosyl diacylglycerol in DNA synthesis in *Synechocystis* sp. PCC 6803. BMC Res Notes 5: 98. 22336148 10.1186/1756-0500-5-98 PMC3311599

[41] Bogos B, Ughy B, Domonkos I, Laczko-Dobos H, Komenda J, et al (2010) Phosphatidylglycerol depletion affects photosystem II activity in *Synechococcus* sp. PCC 7942 cells. Photosynth Res 103: 19–30. 19763873 10.1007/s11120-009-9497-0

[42] Van Mooy BA, Rocap G, Fredricks HF, Evans CT, Devol AH (2006) Sulfolipids dramatically decrease phosphorus demand by picocyanobacteria in oligotrophic marine environments. Proc Natl Acad Sci USA 103: 8607–8612. 16731626 10.1073/pnas.0600540103 PMC1482627

[43] Frentzen M (2004) Phosphatidylglycerol and sulfoquinovosyldiacylglycerol: anionic membrane lipids and phosphate regulation. Curr Opin Plant Biol 7: 270–276. 15134747 10.1016/j.pbi.2004.03.001

[44] Souza V, Eguiarte LE, Siefert J, Elser JJ (2008) Microbial endemism: does phosphorus limitation enhance speciation? Nat Rev Microbiol 6: 559–564. 18521074 10.1038/nrmicro1917

[45] Alcaraz LD, Olmedo G, Bonilla G, Cerritos R, Hernandez G, et al (2008) The genome of *Bacillus coahuilensis* reveals adaptations essential for survival in the relic of an ancient marine environment. Proc Natl Acad Sci USA 105: 5803–5808. 18408155 10.1073/pnas.0800981105 PMC2311347

[46] Juntarajumnong W, Hirani TA, Simpson JM, Incharoensakdi A, Eaton-Rye JJ (2007) Phosphate sensing in *Synechocystis* sp. PCC 6803: SphU and the SphS-SphR two-component regulatory system. Arch Microbiol 188: 389–402. 17541776 10.1007/s00203-007-0259-0

[47] Suzuki S, Ferjani A, Suzuki I, Murata N (2004) The SphS-SphR two component system is the exclusive sensor for the induction of gene expression in response to phosphate limitation in *Synechocystis* . J Biol Chem 279: 13234–13240. 14707128 10.1074/jbc.M313358200

[48] Mizushina Y, Takeuchi T, Hada T, Maeda N, Sugawara F, et al (2008) The inhibitory action of SQDG (sulfoquinovosyl diacylglycerol) from spinach on Cdt1-geminin interaction. Biochimie 90: 947–956. 18343230 10.1016/j.biochi.2008.02.018

[49] Furukawa T, Nishida M, Hada T, Kuramochi K, Sugawara F, et al (2006) Inhibitory effect of sulfoquinovosyl diacylglycerol on prokaryotic DNA polymerase I activity and cell growth of *Escherichia coli* . J Oleo Sci 56: 43–47. 17693698 10.5650/jos.56.43

[50] Elkehal R, Becker T, Sommer MS, Koniger M, Schleiff E (2012) Specific lipids influence the import capacity of the chloroplast outer envelope precursor protein translocon. Biochim Biophys Acta 1823: 1033–1040. 22425965 10.1016/j.bbamcr.2012.02.020

[51] Nowack EC, Vogel H, Groth M, Grossman AR, Melkonian M, et al (2011) Endosymbiotic gene transfer and transcriptional regulation of transferred genes in *Paulinella chromatophora* . Mol Biol Evol 28: 407–422. 20702568 10.1093/molbev/msq209

[52] Horvath I, Glatz A, Nakamoto H, Mishkind ML, Munnik T, et al (2012) Heat shock response in photosynthetic organisms: membrane and lipid connections. Prog Lipid Res 51: 208–220. 22484828 10.1016/j.plipres.2012.02.002

[53] Steiner S, Lori C, Boehm A, Jenal U (2103) Allosteric activation of exopolysaccharide synthesis through cyclic di-GMP-stimulated protein-protein interaction. EMBO J 32: 354–368. 10.1038/emboj.2012.315 PMC3567491 23202856

[54] Matsuda N, Uozumi N (2006) Ktr-mediated potassium transport, a major pathway for potassium uptake, is coupled to a proton gradient across the membrane in *Synechocystis* sp. PCC 6803. Biosci Biotechnol Biochem 70: 273–275. 16428848 10.1271/bbb.70.273

[55] van de Meene AM, Hohmann-Marriott MF, Vermaas WF, Roberson RW (2006) The three-dimensional structure of the cyanobacterium *Synechocystis* sp. PCC 6803. Arch Microbiol 184: 259–270. 16320037 10.1007/s00203-005-0027-y

