# Supplementary figures and images for: Subcellular Localization of Monoglucosyldiacylglycerol Synthase in Synechocystis sp. PCC6803 and Its Unique Regulation by Lipid Environment
Source: PLoS One. 2014 Feb 6;9(2):e88153. doi: 10.1371/journal.pone.0088153 (PMC3916417; doi:10.1371/journal.pone.0088153)

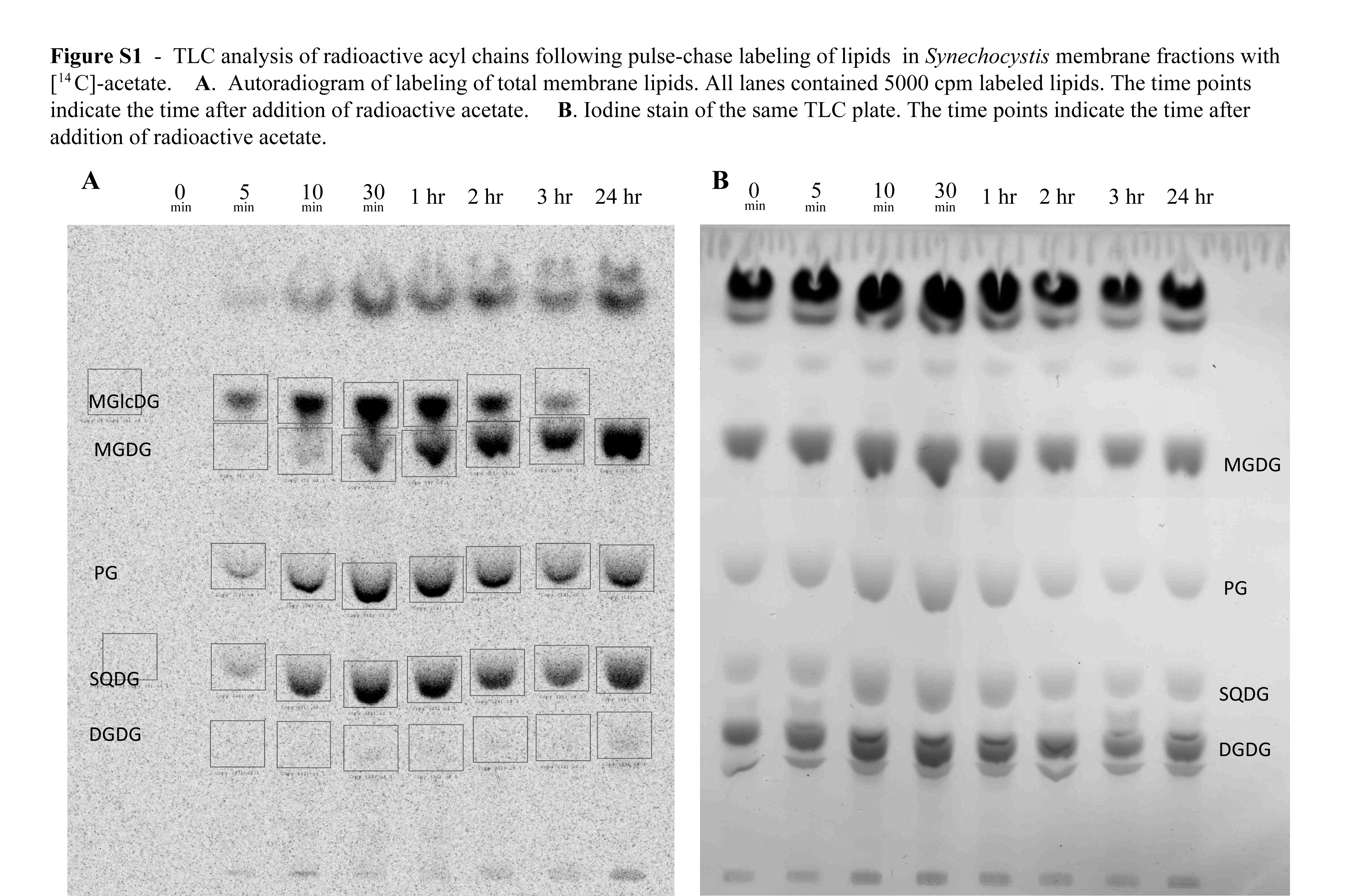

Supplement: Figure S1 — TLC analysis of radioactive acyl chains following pulse-chase labeling of lipids in Synechocystis membrane fractions with [14C]-acetate. (TIF) [file pone.0088153.s001.tif]

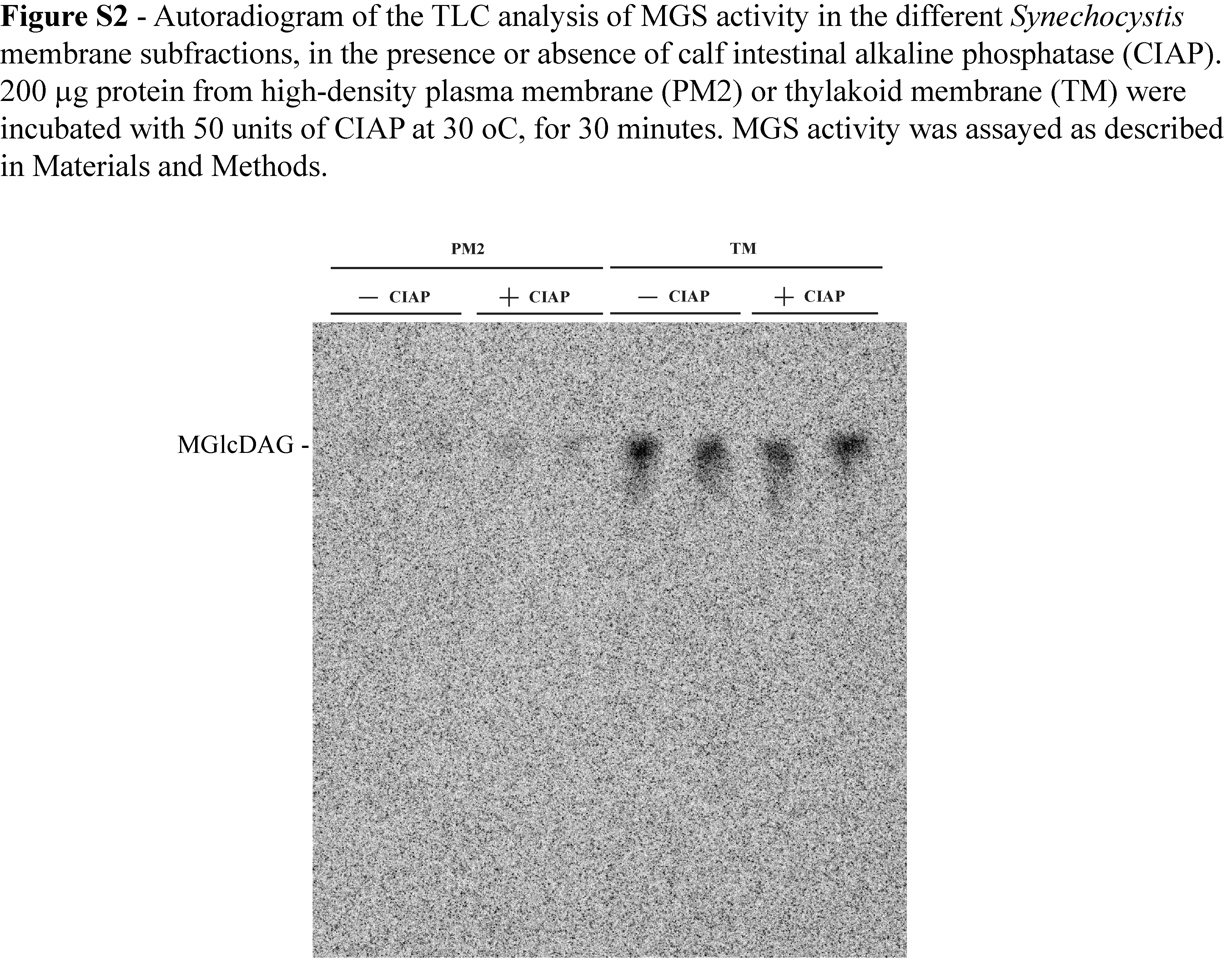

Supplement: Figure S2 — Autoradiogram of the TLC analysis of MGS activity in the different Synechocystis membrane subfractions, in the presence or absence of calf intestinal alkaline phosphatase (CIAP). (TIF) [file pone.0088153.s002.tif]

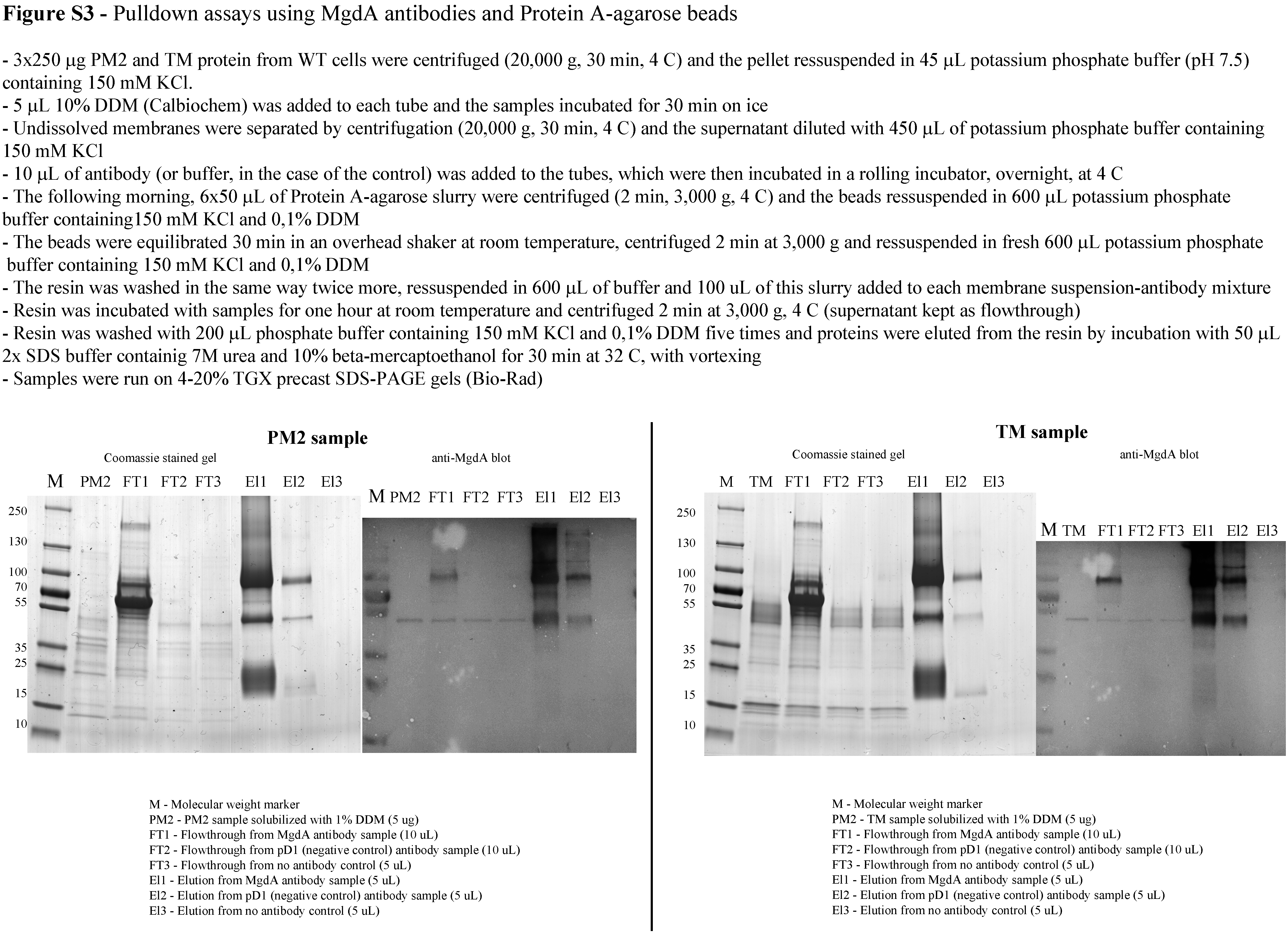

Supplement: Figure S3 — Pulldown assays using MgdA antibodies and Protein A-agarose beads. (TIF) [file pone.0088153.s003.tif]

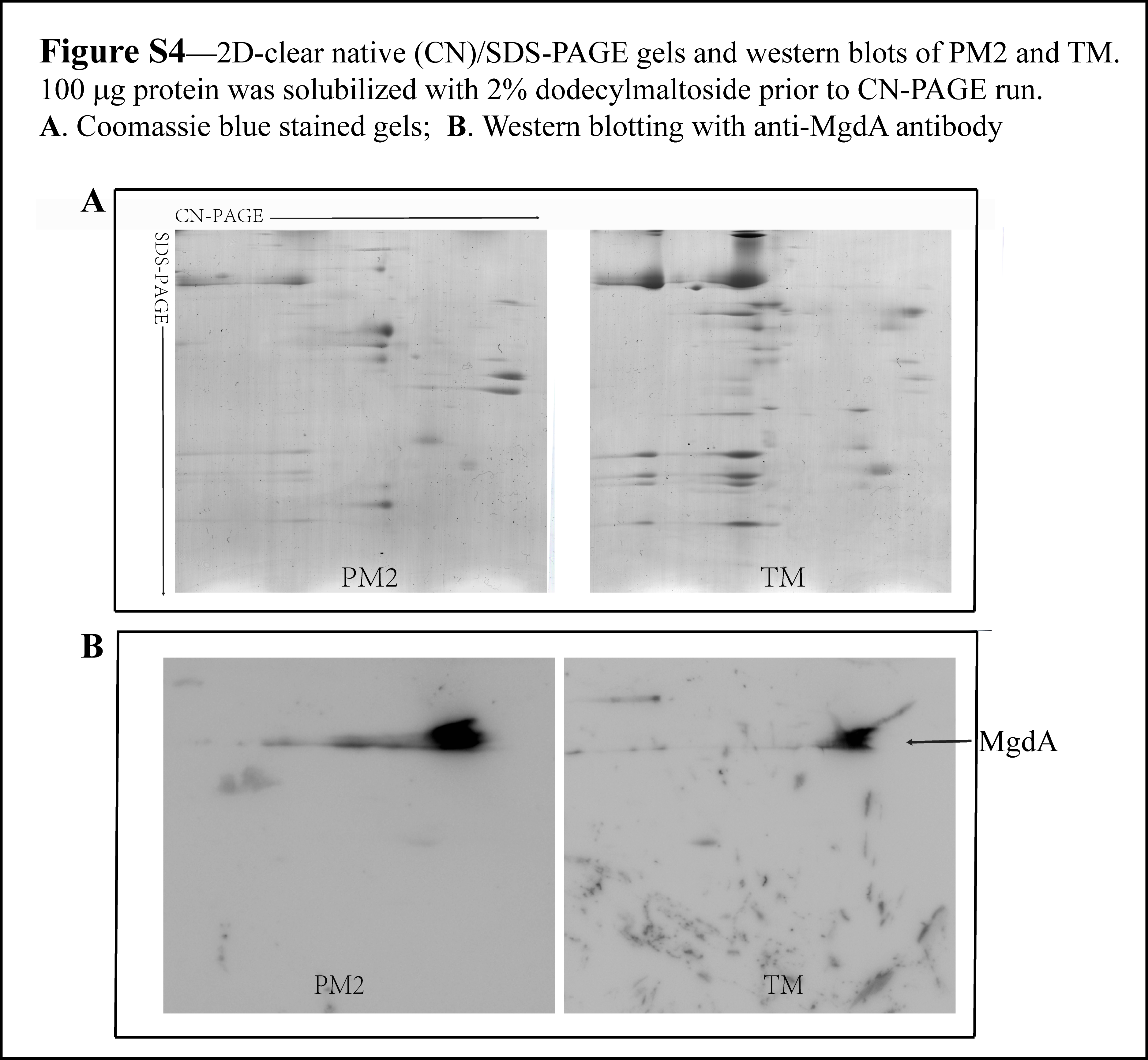

Supplement: Figure S4 — 2D-clear native (CN)/SDS-PAGE gels and western blots of PM2 and TM. (TIF) [file pone.0088153.s004.tif]

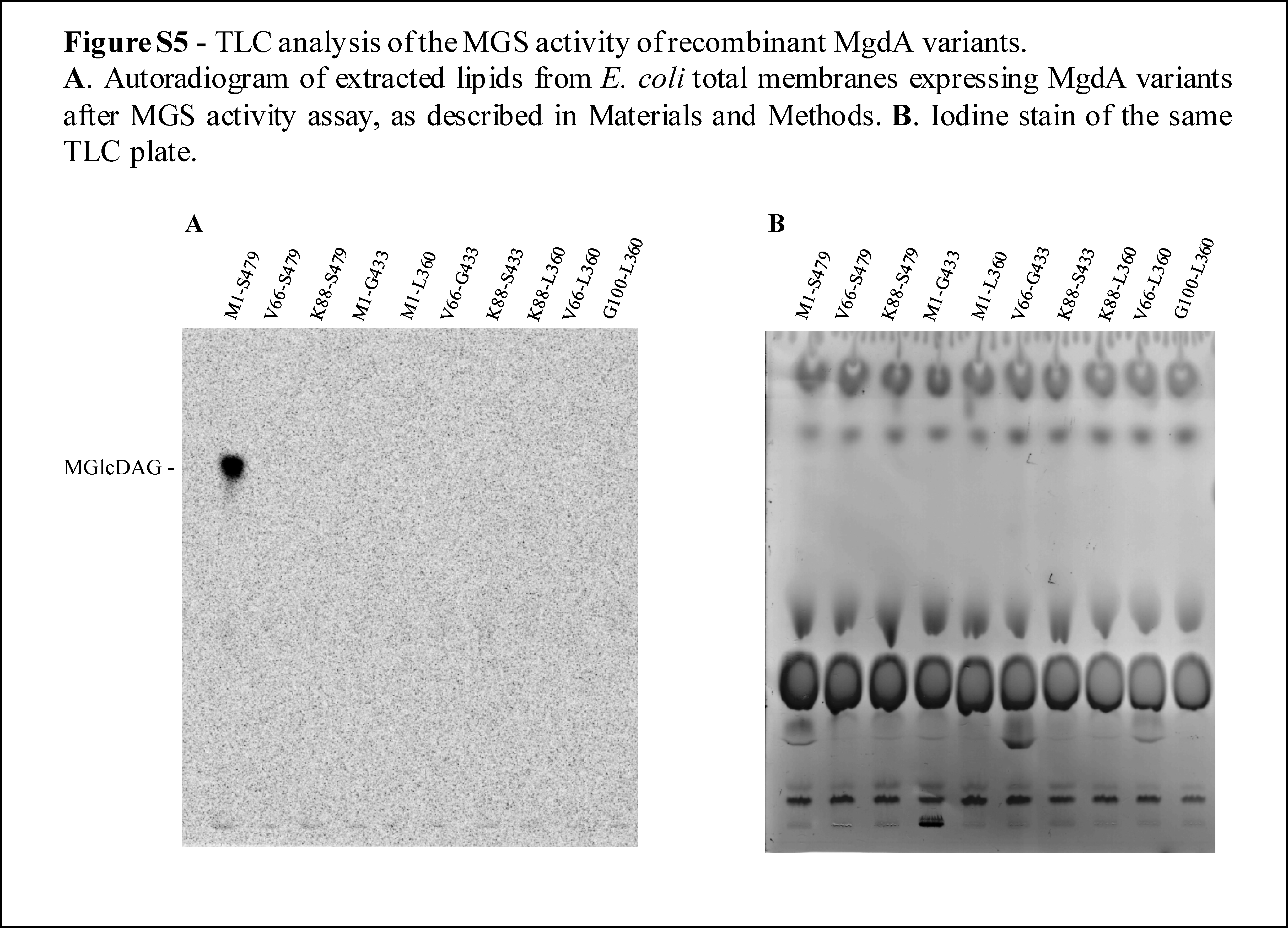

Supplement: Figure S5 — TLC analysis of the MGS activity of recombinant MgdA variants. (TIF) [file pone.0088153.s005.tif]
