# Supplementary material for: Subcellular Localization of Monoglucosyldiacylglycerol Synthase in Synechocystis sp. PCC6803 and Its Unique Regulation by Lipid Environment
Source: PLoS One. 2014 Feb 6;9(2):e88153. doi: 10.1371/journal.pone.0088153 (PMC3916417; doi:10.1371/journal.pone.0088153)
Supplement: Table S1 — List of MGS constructs, the transmembrane helices (TMH) encoded by each construct and the primer sequences used for amplification. (DOCX) [file pone.0088153.s006.docx]

| Clone | Forward primer sequence | Reverse primer sequence | TMH |
| --- | --- | --- | --- |
|  |  |  |  |
| M1-S479 | 5’-TACTTCCAATCCATGATGCCCCAATTTCCGTGGAA-3’ | 5’-TATCCACCTTTACTGTCAACTTTGTTTCAGCTCAAGGGC-3’ | 1, 2, 3, 4, 5 |
| V66-S479 | 5’-TACTTCCAATCCATGGTTTGGGGCAGTTGGCTGG-3’ | 5’-TATCCACCTTTACTGTCAACTTTGTTTCAGCTCAAGGGC-3’ | 2, 3, 4, 5 |
| K88-S479 | 5’-TACTTCCAATCCATGAAAGCAACCCCAGAAGAAGC-3’ | 5’-TATCCACCTTTACTGTCAACTTTGTTTCAGCTCAAGGGC-3’ | 3, 4, 5 |
| M1-G433 | 5’-TACTTCCAATCCATGATGCCCCAATTTCCGTGGAA-3’ | 5’-TATCCACCTTTACTGTCAACCGATGGTGCGAGCTAGT-3’ | 1, 2, 3, 4 |
| M1-L360 | 5’-TACTTCCAATCCATGATGCCCCAATTTCCGTGGAA-3’ | 5’-TATCCACCTTTACTGTCACAAATCCAACTTTTTCTTCCAG-3’ | 1, 2 |
| V66-G433 | 5’-TACTTCCAATCCATGGTTTGGGGCAGTTGGCTGG-3’ | 5’-TATCCACCTTTACTGTCAACCGATGGTGCGAGCTAGT-3’ | 2, 3, 4 |
| K88-G433 | 5’-TACTTCCAATCCATGAAAGCAACCCCAGAAGAAGC-3’ | 5’-TATCCACCTTTACTGTCAACCGATGGTGCGAGCTAGT-3’ | 3, 4 |
| K88-L360 | 5’-TACTTCCAATCCATGAAAGCAACCCCAGAAGAAGC-3’ | 5’-TATCCACCTTTACTGTCACAAATCCAACTTTTTCTTCCAG-3’ | - |
| V66-L360 | 5’-TACTTCCAATCCATGGTTTGGGGCAGTTGGCTGG-3’ | 5’-TATCCACCTTTACTGTCACAAATCCAACTTTTTCTTCCAG-3’ | 2 |
| G100-L360 | 5’-TACTTCCAATCCATGGGAGATGCATCCACGGTGC-3’ | 5’-TATCCACCTTTACTGTCACAAATCCAACTTTTTCTTCCAG-3’ | - |

**Table S1 – List of MGS constructs, the transmembrane helices (TMH) encoded by each construct and the primer sequences used for amplification.** All constructs contained an N-terminal (His)_6_ tag, followed by a site for TEV protease cleavage immediately adjacent to this N-terminus. Transmembrane segments predicted by the OCTOPUS server: 1 – amino acid residues 46-66; 2 – 68-88; 3 – 361-381; 4 – 389-409; and 5 – 435-455.
